# Right Ventricular Strain by Echocardiography: Current Clinical Applications and Future Directions for Mechanics Assessment of the Forgotten Ventricle

**DOI:** 10.3390/jpm15060224

**Published:** 2025-05-30

**Authors:** Mahmoud Abdelnabi, Ramzi Ibrahim, Hoang Nhat Pham, Bobbi Jo Heon, George Bcharah, Girish Pathangey, Milagros Pereyra Pietri, Juan M. Farina, Ian C. Chang, Reza Arsanjani, Chadi Ayoub

**Affiliations:** 1 Department of Cardiovascular Medicine, Mayo Clinic, Phoenix, AZ 85054, USA; abdelnabi.mahmoud@mayo.edu (M.A.); ibrahim.ramzi@mayo.edu (R.I.); heon.bobbi@mayo.edu (B.J.H.); pathangey.girish@mayo.edu (G.P.); pereyra.milagros@mayo.edu (M.P.P.); farina.juanmaria@mayo.edu (J.M.F.); arsanjani.reza@mayo.edu (R.A.); 2 Department of Medicine, University of Arizona, Tucson, AZ 85719, USA; 3 Department of Cardiovascular Medicine, Mayo Clinic, Rochester, MN 55905, USA; 4 Mayo Clinic Alix School of Medicine, Phoenix, AZ 85054, USA; bcharah.george@mayo.edu

**Keywords:** right ventricle dynamics, speckle-tracking echocardiography, two-dimensional echocardiography, three-dimensional echocardiography, heart failure, cardiomyopathies, coronary artery disease, myocardial infarction, pulmonary hypertension, pulmonary embolism, congenital heart diseases, valvular heart diseases, artificial intelligence, cardio-oncology

## Abstract

Myocardial deformation imaging has emerged as a valuable clinical tool for assessing right ventricular (RV) systolic function, providing additional diagnostic and prognostic insights compared to traditional indices of RV function. Two-dimensional speckle-tracking echocardiography is currently the standardized method of choice for measuring RV longitudinal strain (RVLS) in clinical practice. RVLS provides a more sensitive indicator of subtle myocardial dysfunction than conventional parameters for RV function assessment (i.e., tricuspid annular plane systolic excursion, tissue Doppler systolic velocity, fractional area change, or RV ejection fraction), with utility for the risk stratification and surveillance of conditions affecting the right heart. However, accurate interpretation of RVLS requires a comprehensive understanding of RV mechanics, pathology, and loading conditions across various cardiovascular conditions, as well as the effects of image quality and technical aspects of image acquisition and tracking in RV strain measurements. This review provides an updated overview of current practical guidelines for RV strain analysis, current clinical applications, and future directions for its potential use in clinical practice.

## 1. Introduction

Cardiac strain imaging has emerged as a sensitive and reproducible measure of myocardial function, adding incremental clinical value to traditional measures by transthoracic echocardiography (TTE). Strain assessment has also been referred to as ‘speckle imaging’, ‘mechanics assessment’, or ‘deformation imaging’. Strain by speckle-tracking echocardiography is an assessment of myocardial deformation and the change in length between different speckles through the heart’s contraction and relaxation phases. Two-dimensional speckle-tracking echocardiography (2D-STE) has become a widely used imaging technique for assessing myocardial deformation [1]. Strain measurement via tissue Doppler imaging has been used in the past, and 3D strain imaging is likely to become more widely applied in the future.

Strain imaging has become a standard of practice for assessing the left ventricle (LV) in various conditions, with the most evidence in the literature to support its use. Global longitudinal strain (GLS) is a sensitive echocardiographic parameter used to evaluate LV systolic function and assess for subclinical abnormalities before LV ejection fraction (LVEF) becomes abnormal. The pattern on strain imaging can help identify specific disease conditions. Left atrial (LA) strain, in particular LA reservoir strain, is gaining increasing recognition as a marker of atrial function and overall diastolic dysfunction. Similarly, the assessment of the right ventricle (RV) by strain has emerged as a useful diagnostic and prognostic parameter for evaluating RV function and diseases affecting the right heart.

RV assessment is vital in evaluating cardiovascular diseases and patient outcomes. However, its unique anatomy, adaptive remodeling, and functional characteristics present significant challenges for echocardiographic imaging [2]. As the RV wraps around the LV, it can be challenging to visualize by TTE. Parameters used to assess RV function are often one-dimensional and do not consider the entire ventricular geometry. RV longitudinal strain (RVLS), assessed with 2D-STE, has emerged as a valuable method for detecting early RV dysfunction and predicting clinical outcomes, compared to traditional echocardiographic parameters such as RV fractional area change (RVFAC), tricuspid annular plane systolic excursion (TAPSE), and the systolic velocity of the tricuspid annulus (S′) [3]. This review investigates the current evidence, clinical applications, and future directions of RV strain imaging using echocardiography.

## 2. Current Strain Applications for the Left Ventricle and Left Atrium

GLS is more sensitive than left ventricular ejection fraction (LVEF) for detecting subtle systolic dysfunction in patients suspected of having heart failure with preserved ejection fraction (HFpEF) [4]. The American Society of Echocardiography (ASE) recommends GLS for the routine monitoring of myocardial function in patients undergoing potentially cardiotoxic chemotherapy to detect early subclinical LV dysfunction before a decline in LVEF occurs, with a relative reduction of ≥15% from baseline considered clinically significant [5]. Early detection of cardiac dysfunction through GLS enables a timely initiation of cardioprotective therapies, reducing the incidence of cancer therapy-related cardiac dysfunction (CTRCD) and improving long-term outcomes [6,7,8].

GLS is also valuable for identifying early systolic dysfunction in patients with valvular heart disease, providing additional prognostic information that may assist with management [9]. LV strain is well established in amyloid cardiomyopathy as part of the diagnostic evaluation, with significant prognostic value [10]. In other cardiomyopathies, including hypertrophic cardiomyopathy (HCM) and dilated cardiomyopathy (DCM), GLS may detect subclinical LV dysfunction and predict adverse outcomes, including acute heart failure hospitalization and overall mortality [11,12]. Some data suggest that GLS can also help diagnose myocardial ischemia and guide treatment in ischemic heart disease [4].

Left atrial strain (LAS) is an emerging echocardiographic parameter that can reflect the LA’s reservoir, conduit, and contractile functions, providing clinical insights into various cardiovascular diseases that may result in increased LV filling pressures. Its impairment correlates with the degree of diastolic dysfunction. Impaired LAS was associated with higher rates of rehospitalization and cardiovascular death in acute heart failure patients [13]. LAS has also been suggested to have utility for other cardiac processes. LAS was superior compared to other echocardiographic parameters in predicting adverse clinical outcomes, including mortality and heart failure hospitalization in moderate to severe aortic stenosis with preserved LVEF patients [14]. In patients with chronic coronary syndrome and normal LVEF, reduced LAS was associated with a higher risk of adverse cardiovascular events, including new-onset atrial fibrillation and heart failure hospitalization [15]. LAS, particularly LA conduit strain, was a strong independent predictor of adverse outcomes in dilated cardiomyopathy compared to LVEF and LV-GLS [16]. Additionally, LAS provided an incremental prognostic value for assessing major adverse cardiac events in ST-Segment Elevation Myocardial Infarction (STEMI) patients [17].

## 3. RV Strain Analysis

2D-STE is the standard imaging method for assessing RV and LV strain due to its reduced angle dependency, feasibility, and reproducibility compared to tissue Doppler imaging (TDI)-derived strain and strain rate. Although strain measurements derived from TDI using velocity gradients have higher temporal resolution, they are more angle-dependent and require separate analysis for each region of interest. 3D-STE is an emerging technique for strain. It is the least angle-dependent, permitting multidirectional area strain analysis. However, it requires a 3D probe, has the lowest temporal and spatial resolution, and requires further validation before it becomes widely used clinically [18].

RV strain can be classified into RV longitudinal strain (RVLS), circumferential strain, and radial strain, depending on the direction of myocardial speckle movement. RVLS represents a systolic shortening of the myocardial fibers, contributing to about 80% of RV function [3,19]. Data suggest RVLS abnormalities can be detected earlier in RV systolic dysfunction compared to RV circumferential and radial strains [20]. 2D-STE enables the quantification of detailed RV deformation, the detection of early subclinical dysfunction, and comprehensive diagnostic and prognostic insights into various cardiovascular diseases [21]. The European Association of Cardiovascular Imaging (EACVI) and the American Society of Echocardiography (ASE) have developed consensus documents and guidelines to standardize RV strain imaging for clinical practice and scientific research [22,23].

## 4. How to Acquire and Measure RV Strain

**1.**  **Image Acquisition:**

**Figure 1:**
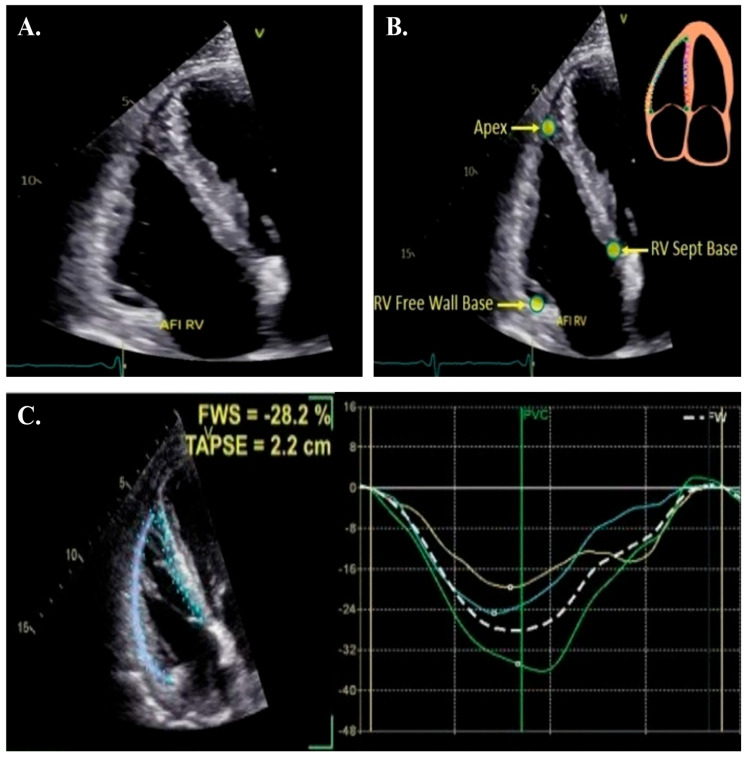
Steps for image acquisition and analysis of RVLS. Panel (**A**): Obtain an optimized RV-focused view from the apical window with all RV free wall segments within the sector. Panel (**B**): Define points for the region of interest (ROI). Panel (**C**): Review segments and adjust ROI to ensure appropriate tracing of RV myocardial segments.

**Figure 2:**
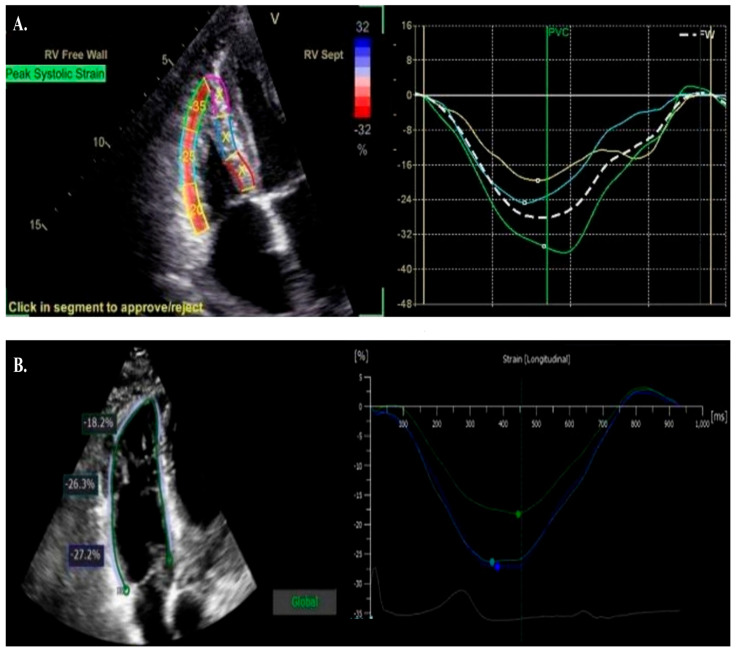
Different RV strain acquisition software packages. Panel (**A**) demonstrates RV strain acquisition using GE Software, with the septal segments blanked out (X Marks) to remove from RVLS analysis. Panel (**B**) demonstrates RV free wall strain visualization using Phillips Software.

Optimal RV-focused apical four-chamber views are essential for adequate visualization of the RV free wall and to avoid the foreshortening of the RV apex compared to the conventional apical four-chamber view [24]. The following steps are recommended for optimal image acquisition (Figure 1 and Figure 2) [21]:

- The patient should be in a steep lateral position with the probe moved laterally and tilted toward the liver to center the LV apex in the scanning sector. This ensures that only the interatrial septum is displayed, avoiding the LV outflow tract or coronary sinus, which enables clear visualization of the largest RV width, apex, and free wall throughout the cardiac cycle, facilitating optimal STE tracking. Optimal image quality requires adjustments to image depth (decreased), width (narrowed), sector angle, and gain (usually needs to be turned down), with a temporal resolution of 50–80 frames per second (higher for higher heart rates). The entire RV myocardium must be included in the image sector throughout the entire cardiac cycle.
- Respiratory maneuvers are often required to enhance spatial resolution, and three consecutive cardiac cycles should be acquired during a breath-hold. Acquiring 3–5 beat cycles is recommended for the optimal cycle to be chosen. Ectopic beats should be avoided.
- RV end-systole is identified using the pulmonary valve closure click (PVC) observed on the RV outflow tract Doppler tracing, which should be acquired immediately afterward to minimize variations between cardiac cycles. RV end-systole is identified via the pulmonary valve closure click (PVC) on RV outflow Doppler tracing, acquired immediately to reduce cycle variation.

**2.**  **Segmental Analysis:**

For a comprehensive assessment, the RV is divided into six segments (the basal, middle, and apical segments of both the RV free wall and the interventricular septum). Free Wall RV longitudinal strain (RVLS) represents the average strain value of the basal, middle, and apical segments of the RV free wall, while RV global longitudinal strain (RVGLS) represents the average strain of all six segments [22]. RVGLS can be affected by left ventricular (LV) systolic function due to the involvement of the interventricular septum (IVS); therefore, current guidelines recommend using RVGLS for RV assessment in clinical practice, along with standardized reference values [21,23].

**3.**  **Potential Errors and Adjustments:**

**Figure 3:**
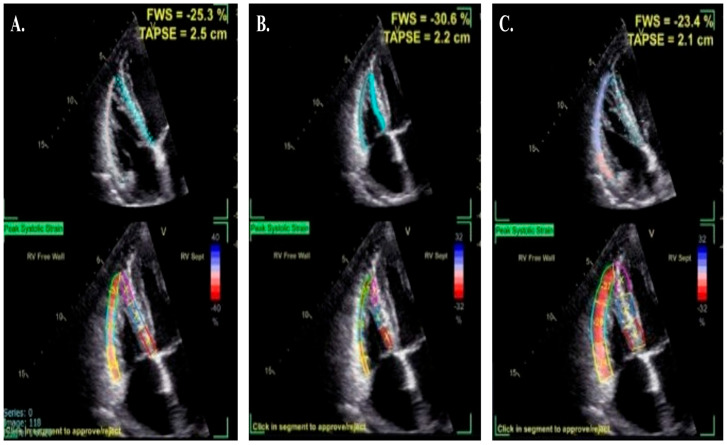
Potential ROI errors in RV strain assessment. Panel (**A**) demonstrates an appropriate RV free wall tracing with a correct strain measurement in the normal range (RVLS: −25.3%). Panel (**B**) shows the effect of setting the ROI too narrowly, resulting in an overestimation of the true RVLS value (−30.6%). Panel (**C**) shows an ROI tracing set too far outward, including the pericardium, with a resulting underestimation of the true RVLS value in the mildly impaired range (−23.4%).

It is essential that even when measuring RVLS only at the free wall, the region of interest (ROI) should include both the RV free wall and IVS. The ROI width must be adjusted to match the thickness and position of the RV free wall myocardium. Incorrect placement, such as extending too far outward (including the pericardium) or too low (below the tricuspid annulus), can underestimate RVLS, while a too-narrow ROI focusing on the endocardial layer may overestimate it (Figure 3) [22]. Different software algorithms can produce differing values depending on whether they measure the endocardium, full wall, or mid-myocardium. Tracking quality should always be visually confirmed using cine loops and segmental strain curves. Suboptimal tracking can often be improved by manually re-adjusting the ROI position. Re-acquisition of the RV-focused apical four-chamber views may be needed [21].

## 5. Reference Ranges and Regional Differences of Right Ventricular Longitudinal Strain

Previous studies investigating reference values for RV strain using 2D-STE showed that the absolute values of RVLS were consistently higher than those of RVGLS, with the lower limit of normal (LLN) for RVLS ranging from −13.3% to −22.7% [25,26,27,28,29]. A practical guide, supported by the 2015 EACVI/ASE chamber quantification recommendations, 2025 ASE guidelines for RV echocardiographic assessment, and the multinational WASE study, suggests that RVLS < −20% is considered abnormal [23,29,30]. Sex differences were noted, with women having higher absolute RVLS values than men by approximately 2±4 strain% % units. LLN for RVLS was about 1% higher in women, with no significant ethnic differences observed in the WASE study [25,27,31].

A meta-analysis by Wang et al. identified a normal pooled mean of −26.9% (−28.0%, −25.9%) by echocardiography in healthy subjects for RVLS [32]. Although normal values may vary between software and vendors used in different laboratories, the scale used in our laboratory was as follows: normal: ≤−25%; mildly reduced: ≤−20% to −25%; moderately reduced: ≤−15% to −20%; and severely reduced: < −15%. Due to potential inter-vendor variability, it is important that reference values specific to an institution’s laboratory are applied. Age-related decline in RVLS has been suggested to be minor and not clinically significant in adults. However, children have higher RVLS values, with an LLN of −27% for RVLS; therefore, applying a uniform LLN in children may not be appropriate [25,31,33]. Unlike LV strain, where the bull’s-eye strain map can facilitate qualitative assessment in different disease processes, such as differentiating amyloidosis from apical or septal hypertrophic cardiomyopathy or in assessing for regional wall motion abnormalities, RV strain remains predominantly quantitative (e.g., applying free wall RVLS). Future studies are needed to develop qualitative RV assessment methods [1,23]. Given the known inter-vendor variability, it is recommended that the same vendor be used for serial strain comparisons. Nonetheless, future research should focus on developing standardized strain measurement methods and reference ranges to enable comparability across different vendors [1,23]. (Table 1 summarizes current published data for RV strain reference values.)

## 6. Clinical Applications of RV Strain

**Figure 4:**
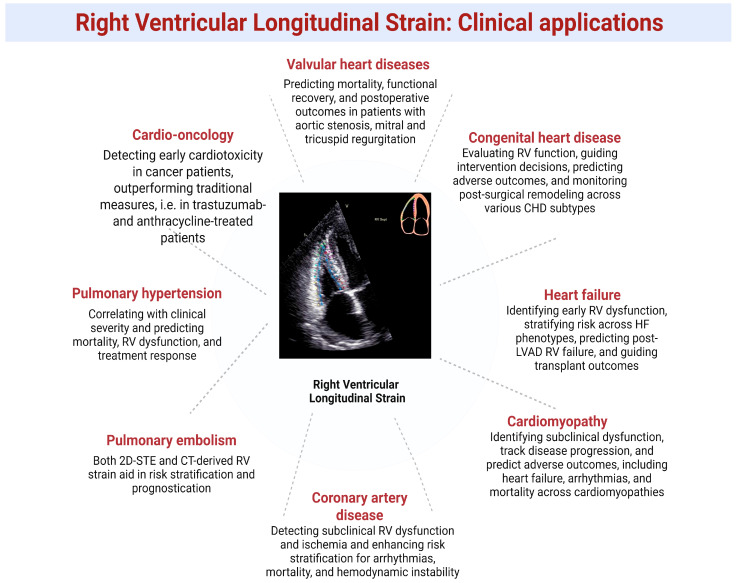
Summary of clinical applications of RV strain.

RVLS provides additional functional insights and prognostic value beyond conventional echocardiographic indices of RV systolic function across various cardiovascular diseases (Figure 4).

## 7. Right Ventricular Strain in Heart Failure

In the early stages of heart failure and in heart failure with preserved ejection fraction (HFpEF) with LVEF ≥ 50%, impaired LV diastolic dysfunction is the primary manifestation. In both HFpEF and heart failure with reduced ejection fraction (HFrEF), with LVEF < 50%, there may be associated RV dysfunction [39,40]. RV dysfunction in these settings may be driven by complex pathophysiologic mechanisms:

**1.**  **Myocardial Injury:** Comorbidities such as coronary artery disease, hypertension, diabetes mellitus, obesity, and chronic obstructive pulmonary disease, among others, contribute to systemic pathways of myocardial damage promoting RV myocardial hypertrophy and fibrosis resulting in RV dysfunction [41].
**2.**  **Atrial Fibrillation:** Atrial fibrillation shortens ventricular filling time, elevates left atrial pressure, and increases pulmonary pressures, leading to greater RV afterload and dysfunction [41].
**3.**  **Interventricular Interaction:** The functional interdependence between the LV and RV, mediated through the shared interventricular septum and LV contraction contributing 20–40% of RV contractile force, leads to RV dysfunction with LV dysfunction [42].
**4.**  **Pulmonary Hypertension:** Chronic pulmonary venous hypertension due to LV systolic dysfunction increases RV afterload, impairing its function [41]. The RV may be affected in all types of pulmonary hypertension.

- **Acute heart failure**

Previous studies have studied the effects of RV function on clinical outcomes in acute and chronic heart failure, affecting management strategies and long-term prognosis [41,43]. In a retrospective analysis of 618 patients with acute HF, impaired RVLS was an independent predictor of adverse outcomes, demonstrating incremental prognostic value beyond clinical and conventional echocardiographic parameters [44]. Borovac et al. [45] found a strong correlation between RVLS and conventional RV functional indices, such as tricuspid annular plane systolic excursion (TAPSE), RV fractional area change (FAC), and RV systolic velocity (S′), in patients with acute HF exacerbations. Additionally, Park et al. [37] reported in a cohort of 1824 patients with acute HF that reduced RVGLS was significantly associated with all-cause mortality, with the worst outcomes in those with reduced biventricular strain. In critically ill patients, RV subcostal strain was highly correlated with apically acquired strain, suggesting it is a viable alternative when subcostal views are more feasible from a technical acquisition perspective [46].

- **Heart failure with reduced ejection fraction**

RV dysfunction is frequently observed in patients with HF with reduced ejection fraction (HFrEF). Houard et al. [13] demonstrated that RVLS and RVGLS predicted overall and cardiovascular mortality in 266 patients with HFrEF, providing incremental prognostic value over cardiac magnetic resonance (CMR) parameters, including CMR-RVEF, CMR-RVGLS, TAPSE, and FAC [47]. Similarly, in 332 stable HFrEF outpatients, RVLS and RVGLS independently predicted all-cause mortality, cardiovascular death, heart transplantation, and/or death due to HF worsening [42]. Impaired RVGLS was associated with higher New York Heart Association (NYHA) class, larger LV volumes, reduced LVEF, and impaired LV diastolic and RV systolic function. RVGLS ≥ −14.8% independently predicted long-term adverse events after adjusting for clinical and echocardiographic factors, providing incremental prognostic value beyond LV function in a study of 171 patients with HFrEF (LVEF ≤ 35%) [48]. Additionally, in a study of 288 stable HFrEF outpatients, Carluccio et al. found that RVLS and RVGLS were associated with worse outcomes. However, RVLS remained an independent predictor of adverse events after adjusting for clinical and echocardiographic factors, including LV strain, indicating superior prognostic value over RVGLS.

- **Heart failure with preserved ejection fraction**

RV dysfunction is common in heart failure with preserved ejection (HFpEF), affecting at least 20% of patients, with prevalence reaching up to 50% [49,50,51]. A study of 86 HFpEF patients showed that RVLS, assessed by 2D-STE, decreased progressively with worsening LV diastolic dysfunction (grades 1 through 3). RVLS was moderately associated with diastolic function and weakly correlated with age, BNP, RVFAC, and TAPSE, highlighting its sensitivity for detecting early RV systolic dysfunction in mild-to-moderate diastolic dysfunction [52]. In a study of 201 HFpEF patients, Morris et al. found RVGLS was lower compared to asymptomatic diastolic dysfunction patients, with 75% showing RV longitudinal dysfunction. Reduced RVGLS was significantly associated with a worse NYHA functional class [53]. Additionally, Lejeune et al. found that 19% of HFpEF patients had reduced RVGLS (<−17.5%), which was significantly worse than controls. RVGLS was an independent predictor of overall mortality and HF rehospitalization compared to conventional echocardiographic parameters and had a stronger correlation with CMR-derived RVEF, providing a more accurate assessment of RV function [54].

- **Left Ventricular Assist Devices**

RV failure after LV assist device (LVAD) implantation remains a cause of increased morbidity and mortality [55,56,57]. Previous studies have suggested that RVLS may assist in risk stratification in patients undergoing LVAD implantation. Dufendach et al. [15] demonstrated that reduced RVLS before LVAD implantation strongly predicted post-implantation RV failure, with a c-index of 0.65 at a cutoff of −5.64%. In contrast, RVGLS was not predictive, as it is primarily influenced by the interventricular septum [58]. Another study showed that RVLS was more strongly associated with RV failure after LVAD implantation than TAPSE or the Michigan risk score, suggesting independent and incremental value in predicting RV failure [59]. Similarly, a meta-analysis of 4428 patients who underwent LVAD implantation identified 2D RVLS as having the strongest predictive effect for post-LVAD RV failure [60].

- **Cardiac Transplantation**

Several studies have demonstrated the prognostic value of RV strain parameters, guiding risk stratification in heart transplant recipients. Barakat et al. studied the predictive utility of RVLS and LVGLS in low-risk heart transplant recipients at 1-year post-transplant, concluding that RVLS and RV-FAC were independently associated with adverse outcomes such as overall mortality, coronary allograft vasculopathy, and rejection [61]. Similarly, Ji et al. investigated the feasibility and prognostic implications of RV longitudinal shortening fraction (RVLSF) in heart transplant recipients, concluding that RVLSF and RVLS were independently associated with poor prognosis [62]. Moreover, Tian et al. used 3D-STE in heart transplant candidates, showing that 3D-RVLS correlated with severe myocardial fibrosis (MF) and had a better predictive value than 2D-RVLS and conventional RV echocardiographic parameters [63].

RV strain assessment provides incremental diagnostic and prognostic value beyond traditional echocardiographic measures across the spectrum of heart failure, guiding management strategies and long-term outcomes.

## 8. Right Ventricular Strain in Cardiomyopathies

RV dysfunction is a significant predictor of adverse outcomes in different types of cardiomyopathies [64,65]. Studies have assessed RV strain in several types of cardiomyopathies, particularly arrhythmogenic right ventricular cardiomyopathy (ARVC), hypertrophic cardiomyopathy (HCM), nonischemic dilated cardiomyopathy (NIDCM), and amyloidosis.

**1.**  **Arrhythmogenic Right Ventricular Cardiomyopathy (ARVC)**

Studies investigating RV strain in ARVC have demonstrated its utility in diagnosing and managing ARVC, providing additional benefits over traditional imaging techniques. There are a number of studies assessing the diagnostic utility of RV strain by CMR [66,67,68,69]. In terms of echocardiography, Prakasa et al. identified peak RV systolic velocity < 7.5 cm/s and RVLS < 18% as the best parameters for ARVC diagnosis, with sensitivities of 67% and 73% and specificities of 89% and 87%, respectively, compared to controls [70]. Meanwhile, Malik et al. showed that RVLS is significantly associated with the rate of structural progression in ARVC patients. Specifically, patients with RVLS < −20% had a higher risk of structural progression, indicating its potential utility in identifying patients who require closer follow-up [71]. Namasivayam et al. also highlighted the added diagnostic value of RVLS to standard echocardiographic measures, identifying high-risk patients with ARVC, whereas LV strain was additive in diagnostic value [72].

Moreover, Jacquemyn et al. reported that impaired atrial and ventricular strain, including RVLS, were significantly associated with the development of HF in ARVC patients, with strain values below the normal limit being associated with an eight-fold increase in HF risk patients [73]. Anwer et al. demonstrated that impaired RVGLS and right atrial GLS (−11.5% and 22.8%, respectively) are independent predictors of adverse cardiovascular events, sustained ventricular arrhythmia, and cardiovascular death in ARVC patients during a median follow-up of 4.9 years [74]. As such, RV strain imaging can aid in diagnosis, risk stratification, and management in ARVC.

**2.**  **Hypertrophic Cardiomyopathy (HCM)**

In HCM patients, D’Andrea et al. used exercise STE to demonstrate impaired RV contractile reserve. This impairment was associated with diminished exercise capacity and subclinical myocardial damage [75]. Hiemstra et al. reported that RV dysfunction, as measured by RVLS, is relatively frequent in HCM patients and associated with adverse outcomes, including all-cause mortality and heart failure development. Specifically, impaired RV four-chamber longitudinal strain (RV4CLS) and RVLS were associated with worse survival outcomes [76]. Additionally, Mahmod et al. found that RV function, including RV strain, declines over time in HCM patients, even when LVEF is preserved. This decline in RV function was associated with adverse cardiovascular outcomes, such as non-sustained ventricular tachycardia (NSVT) and composite cardiovascular events [77]. Moreover, Qian et al. studied RV global strain techniques in HCM patients with and without RV hypertrophy. RVGLS and global circumferential strain were independent predictors of adverse events, providing additional prognostic value [78].

**3.**  **Nonischemic dilated cardiomyopathy (NIDCM)**

In patients with NIDCM, Vîjîiac et al. showed that RVLS, 2D-RVGLS, and 3D-RVEF were significantly impaired and associated with adverse events. 2D-RVGLS and 3D-RVEF independently predicted outcomes after adjusting for age and NYHA class. Although 2D-RVGLS was not independently related to adverse events after adjusting for LV diastolic dysfunction, it remained useful in detecting subtle RV dysfunction [79]. Another study demonstrated that RVFAC, TAPSE, and 2D-RVLS independently predicted prognosis, with a combined RVFAC and RVLS assessment offering better risk stratification in high-risk NICM patients [80].

**4.**  **Amyloidosis**

RV strain can have a useful role in the diagnosis and prognosis of cardiac amyloidosis, including both light-chain (AL) and transthyretin (ATTR) amyloidosis. RVLS by echocardiography is impaired in patients with cardiac amyloidosis, and relative sparing of the RV apical segment can also be observed in the RV free wall [81]. RV strain is independently associated with poor outcomes in cardiac amyloidosis. Studies have shown that RVLS is a strong predictor of all-cause mortality in AL amyloidosis [82]. Similarly, in transthyretin amyloidosis, RVLS has been shown to provide incremental prognostic value over traditional echocardiographic parameters and biomarker-based staging systems [83]. Given its diagnostic and prognostic value, RV strain assessment should be integrated into the routine evaluation of patients with suspected or confirmed cardiac amyloidosis to facilitate early diagnosis, risk stratification, and management decisions. Previous data suggest that RVLS values < −16% are associated with significantly worse survival outcomes and can guide therapeutic interventions [84,85].

Given its diagnostic and prognostic significance, integrating RV strain assessment into the routine evaluation of various cardiomyopathies may improve early detection, individualized monitoring, and targeted management strategies in high-risk patients.

## 9. Right Ventricular Strain in Acute Myocardial Infarction and Coronary Artery Disease

Several studies have highlighted RV strain’s diagnostic and prognostic value in acute myocardial infarction (AMI) as an independent predictor of adverse outcomes, including ventricular arrhythmias, sudden cardiac death, and overall mortality. Risum et al. demonstrated that RVLS assessed by 2D-STE was an independent predictor of sudden cardiac death (SCD) and ventricular arrhythmias in post-acute MI patients [86]. Bachmann et al. showed that RVGLS (as well as LV and LA strain) improve mortality prediction in acute MI [87]. Sonmez et al. found decreased RV myocardial velocities, strain, and strain rates in anterior MI, indicating subclinical RV dysfunction [88]. Similarly, Sevimli et al. reported significantly lower RV strain and strain rates in patients with RV infarction, confirming the utility of RV strain in identifying RV involvement [89]. Radwan et al. concluded that RV dysfunction is significant in anterior MI and that strain analysis can detect subclinical dysfunction even without electrocardiographic evidence of RV involvement [90].

Other studies highlight RV strain’s diagnostic and prognostic significance in chronic coronary artery disease (CAD). Chang et al. showed that RVLS was significantly impaired in patients with right coronary artery (RCA) stenosis, particularly proximal involvement, revealing occult RV dysfunction often missed by conventional echocardiographic parameters [91]. Chang et al. further demonstrated that reduced RVLS (≤−18%) predicts CV mortality and ventricular arrhythmias in non-acute coronary syndrome angina [92]. The ASE guidelines comment on the importance of RV function assessment, including RV strain, during stress echocardiography to detect subtle RV ischemia or dysfunction in ischemic heart disease [93]. Although data regarding RV strain assessment in CAD are promising, its implementation in routine clinical practice, especially in AMI, is not completely understood.

## 10. Right Ventricular Strain in Pulmonary Embolism

There is utility of RV strain assessment in acute pulmonary embolism (PE), with prognostic significance. Platz et al. assessed regional RVLS by 2D-STE in acute PE patients, reporting that RV strain was significantly reduced in all regions of the free wall and the mid and basal septum, with the strain rate also markedly reduced in these segments [94]. Vitarelli et al. demonstrated that in patients with acute submassive PE, 3D-RVEF and mid-RVLS were significantly reduced among other echocardiographic parameters compared to controls. These parameters were sensitive predictors of adverse events and were independently associated with poor outcomes [95].

Furthermore, Descotes-Genon et al. compared RV strain values between low- and intermediate-to-high-risk PE patients using 2D strain imaging, demonstrating that global and regional RVLS were significantly reduced in intermediate-to-high-risk PE patients, particularly in the apical and mid segments of the free wall [96]. Additionally, Wilson et al. evaluated RVGLS in intermediate-risk PE patients and showed that reduced RVGLS values were associated with higher 30-day mortality. An RVGLS cutoff value < −17.7 demonstrated high sensitivity and specificity for predicting mortality, outperforming traditional echocardiographic parameters and CT findings [97].

## 11. Right Ventricular Strain in Pulmonary Hypertension

RV strain has a role in pulmonary hypertension (PH) to assess RV function and its clinical and prognostic implications. Li et al. demonstrated that 2D and 3D-RVLS are significantly reduced in pulmonary arterial hypertension (PAH) patients and are superior to circumferential and radial strain in predicting clinical outcomes. RVLS significantly predicts adverse outcomes and can track clinical improvement following vasodilator therapy [98]. Similarly, Smith et al. assessed RV function using 3D-STE in PH patients, concluding that all RV strain parameters, including RVLS, area strain (AS), and circumferential strain (CS), were reduced in PH. However, only AS and CS correlated with RVEF. AS was the strongest independent predictor of all-cause mortality, highlighting its value in assessing RV function and clinical outcomes [99].

Shukla et al. conducted a systematic review and meta-analysis on the prognostic value of RV 2D-STE in a large and mixed population of PH patients, concluding that RV strain parameters were significantly associated with all-cause mortality compared to traditional echocardiographic measures [100]. Hardegree et al. evaluated the role of serial quantitative assessment of RV function by STE strain imaging in PAH patients, showing that improvements in RV strain after PAH therapies were associated with improved clinical outcomes and lower mortality risk [101].

Filusch et al. showed that RV strain and strain rate are impaired in idiopathic PAH patients compared to healthy controls. These parameters correlated with elevated NT-proBNP levels and reduced 6 min walk distance (6-MWD), mean pulmonary artery pressure (mPAP), and pulmonary vascular resistance (PVR), indicating their utility in assessing RV dysfunction and disease severity [102]. Sachdev et al. demonstrated that RVLS and strain rate independently predict future right-sided HF, clinical deterioration, and overall mortality in PAH patients. RVLS < −12.5% had a higher rate of disease progression and mortality risk, and a 5% absolute decline in RVLS was associated with a 2.9-fold higher death rate at 1 year [103]. Ünlü et al. proposed the RVLS to systolic pulmonary artery pressure (RVLS/sPAP) ratio as a new prognostic marker in patients with pre-capillary PAH, showing that it was an independent predictor of clinical outcomes, including mortality and heart–lung transplantation [104].

**Figure 5:**
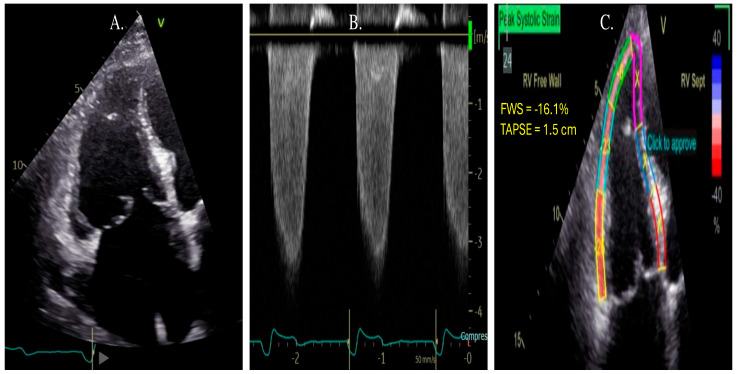
Example of strain analysis in a case of pulmonary hypertension (PH). Panel (**A**) shows an RV-focused view of a dilated RV with a lack of coaptation of the tricuspid valve but with a visual appearance of normal RV systolic function. Panel (**B**) demonstrates a continuous wave Doppler signal of severe tricuspid regurgitation, with RVSP of 51 mmHg. Panel (**C**) demonstrates RV strain analysis with impaired RVLS of −16.1%, which carries incremental prognostic significance in this patient with PH.

In patients with systemic sclerosis, decreased biventricular longitudinal strain has been observed to be primarily due to pulmonary hypertension rather than systemic sclerosis itself, and RVLS has been correlated with mean pulmonary arterial pressure (mPAP) and pulmonary vascular resistance (PVR) [105]. RV strain metrics, including RV-PA coupling ratios, predict adverse clinical outcomes in patients with systemic sclerosis and pulmonary vascular disease. These metrics correlate with invasive pressure–volume loop measurements and can enhance risk stratification [106]. As such, integrating RV strain assessment into routine PH evaluation can improve early diagnosis, risk stratification, and guide therapeutic decision making (Figure 5).

## 12. Right Ventricular Strain in Cardio-Oncology

Cardio-oncology is a rapidly growing field due to improved cancer survival and increasing cancer therapeutics that may adversely affect cardiac function [107]. Cardiovascular imaging remains essential in cardio-oncology. Since the 2014 ASE/EACVI consensus, significant advancements in imaging techniques for cardiotoxicity screening and surveillance have emerged, supported by imaging societies and driven by new antineoplastic therapies [108,109]. According to the American College of Cardiology Cardio-Oncology and Imaging Councils, and the European Society of Cardiology guidelines, cancer treatment-related cardiac dysfunction can be defined as definite with LVEF reduction by ≥10% to a value of <50% or possible with LVEF reduction by ≥10% to a value of 50–55%, or LVEF reduction by <10 percentage points to a value of <50%, or relative reduction in GLS of ≥15% without significant reduction in LVEF [8,107].

In chemotherapy recipients, strain imaging can detect early signs of LV and RV dysfunction before overt clinical symptoms or traditional echocardiographic parameter abnormalities, proving its sensitivity and prognostic value to guide early preventive and therapeutic interventions [110]. Previously published data suggested that RV strain analysis is superior to conventional echocardiographic parameters for early detection of cardiotoxicity in cancer patients or survivors. Keramida et al. reported a percent change of −14.8% (relative reduction) in 2D-RVGLS predicted trastuzumab-induced cardiotoxicity in HER2-positive breast cancer with 66.7% sensitivity, 70.8% specificity, and 90% accuracy [111]. Wang et al. identified a higher prevalence of RV systolic dysfunction in patients with diffuse large B-cell lymphoma (DLBCL) treated with anthracyclines who experienced cardiotoxicity. Additionally, 2D-RVLS and 3D-RVEF are superior to 2D-RVGLS and conventional echocardiographic measures in detecting subtle myocardial dysfunction and predicting patient outcomes [112].

Moreover, in a meta-analysis on anthracycline-induced subclinical RV dysfunction in breast cancer patients, RVGLS and RVLS showed significant reduction post-chemotherapy, indicating RV dysfunction, while traditional parameters such as TAPSE, FAC, and TDI S′ showed less considerable reduction. No significant association was found between LVEF changes and RV strain reductions, indicating RV dysfunction is not solely dependent on LV impairment [113]. RV strain integration into routine cardio-oncology echocardiographic surveillance, in addition to guideline-directed GLS measures, may facilitate the earlier identification of RV dysfunction and risk stratification, as well as timely intervention in cancer survivors.

## 13. Right Ventricular Strain in Valvular Heart Diseases

**1.**  **Aortic Stenosis**

Studies have shown that RV strain has a prognostic value in aortic stenosis (AS). Kitano et al. demonstrated that only LVGLS predicted cardiac events in asymptomatic AS [114], but a prospective study in low-flow, low-gradient AS (LF-LG AS) showed that reduced RVLS (<13%) was independently associated with higher mortality, with stress RVLS adding prognostic value [115]. A surgical aortic valve replacement (SAVR) cohort showed that one-third had RV dysfunction, defined by one or more of the following: TAPSE < 17 mm, RVLS < −20% on TTE, or RVEF < 50%, detected earlier by echocardiography than CMR [116]. While LV strain improved post-AVR, RV strain worsened, suggesting a negative impact of surgery on RV function [117]. RVLS < −17.3% predicted low-cardiac-output syndrome after SAVR, with high sensitivity (86.7%) but moderate specificity (61.7%) [118]. RV strain studies in transcatheter aortic valve replacement (TAVR) and surgical aortic valve replacement (SAVR) highlight its prognostic value with RVLS via 2D-STE predicting 1-year mortality after TAVR [119] with better baseline RV function correlating with functional recovery post-TAVR, with improved RVGLS and TAPSE, while worsened post-SAVR [120]. A high prevalence of RV dysfunction in severe AS patients undergoing TAVR, with significant improvement, was observed one year post-procedure in normal-flow AS cases [121]. Stable RV function post-TAVR but decline post-SAVR, suggesting TAVR benefits patients with pre-existing RV dysfunction, which might be explained by postoperative changes such as pericardial adhesions [122]. RVLS and RV-arterial coupling (TAPSE/sPAP and RVLS/sPAP) were identified as superior risk stratification tools in HF patients undergoing TAVR [123].

**2.**  **Mitral Regurgitation**

RV strain offers prognostic value in mitral regurgitation (MR) by improving the detection of RV dysfunction and guiding clinical decisions. In ischemic MR (iMR), RV strain parameters correlated strongly with CMR-RVEF, surpassing conventional echocardiographic indices in detecting RV dysfunction and nonischemic fibrosis [124]. Impaired RV systolic function has been identified as an independent predictor of postoperative cardiovascular survival in primary MR, with biventricular dysfunction indicating a particularly poor overall prognosis [125]. Frequent RV dysfunction was noted following mitral valve (MV) repair for degenerative MR, particularly in patients with elevated preoperative pulmonary artery systolic pressure (PASP) [126]. RV dysfunction and tricuspid annular dilatation were linked to pulmonary hypertension and left-sided chamber enlargement in severe MR due to MV prolapse, emphasizing the need for comprehensive RV assessment before surgery [127]. Transapical transcatheter mitral valve implantation (TMVI) significantly improved RV function, demonstrated by an increased RV stroke work index (RVSWI), RV fractional area change, and RVLS, along with decreased PASP and tricuspid regurgitation (TR) [128]. In patients with severe iMR undergoing combined MV and tricuspid valve (TV) repair, RV remodeling did not improve postoperatively. Despite favorable LV remodeling and improved LVEF, RV dysfunction progressed with worsening TR recurrence, highlighting the need for optimized surgical strategies for iMR-associated TR [129]. 3D-STE significantly improved LV and RV strain after MitraClip implantation. However, patients with preexisting RV dysfunction showed limited improvement in LV strain, suggesting a need for earlier intervention or alternative therapies [130]. Preserved RV reserve—defined as RV strain improvement one month post-MV repair—was associated with a lower risk of HF hospitalization [131].

**3.**  **Tricuspid regurgitation**

RV strain, particularly RVLS, may have prognostic value in patients with tricuspid regurgitation (TR). RVLS was a superior predictor of mortality compared to conventional echocardiographic parameters, with impaired RVLS independently associated with all-cause mortality in patients with significant functional TR [132]. An incremental prognostic value of RVLS over traditional clinical and imaging risk factors [133]. Similarly, RVLS was identified as the strongest predictor of mortality and HF in isolated severe TR [134]. Additionally, RVLS identified early RV dysfunction in patients with severe TR and normal RV systolic function, enhancing the prediction of HF and overall survival [135]. Furthermore, impaired RV strain and TAPSE added prognostic value for 2-year mortality in patients with functional TR [136]. Additionally, RVLS worse than −24% is associated with a higher risk of postoperative mortality or hospitalization in patients who had isolated surgery for severe functional TR [137]. Given its incremental prognostic value and risk stratification, RV strain assessment should be integrated into valve disease assessment to optimize treatment timing and improve long-term clinical outcomes.

## 14. Right Ventricular Strain in Congenital Heart Diseases

Assessing RV function in congenital heart disease (CHD) is complex due to the dependence of strain values on pre- and afterload and anatomical variations in different pathologies. RV strain has proven prognostic value across CHD subtypes. In atrial septal defect (ASD), RV size and function can guide closure decisions. In atrial septal defect (ASD), RVLS was higher before and lower after ASD closure, correlating with shunt fraction and volume overload, with post-closure reductions attributed to persistently larger RV volumes [138]. In ASD and repaired Tetralogy of Fallot (TOF) with pulmonary regurgitation (PR), similar RV remodeling occurs, but only TOF patients show reduced strain, indicating distinct remodeling processes [139]. In TOF, 2D-RVLS independently predicts CMR-derived RVEF [140]. RVLS also predicted HF, transplantation, arrhythmias, and mortality in Ebstein’s disease and TOF [141] HF and mortality in systemic RV conditions, such as post-atrial switch and congenitally corrected transposition of the great arteries (ccTGA) [141]. Additionally, abnormal STE and CMR LV and RV strain measures in repaired TOF patients with residual PR can detect subclinical biventricular dysfunction despite normal LVEF [142]. In post-percutaneous pulmonary valve implantation (PPVI), RVLS and strain rate via STE strongly correlated with improved exercise capacity [143].

## 15. Future Directions

### 15.1. Three-Dimensional Echocardiography

Emerging data suggest that 3D echocardiography can provide a comprehensive evaluation of RV function, enabling early detection of RV dysfunction. The RV’s complex morphology, with its asymmetrical shape and thin walls, makes it challenging to assess accurately using 2D methods. 3D-STE has emerged as a novel technique to overcome some of the limitations of 2D-STE [144]. Previous studies showed its accuracy and reproducibility in RV function assessment in patients with different conditions, such as cardiac transplant, pulmonary hypertension, and congenital heart diseases [98,145,146].

Moreover, 3D-STE has been shown to correlate well with CMR-derived parameters. Li et al. showed that 3D-STE measurements of RV volumes, RVEF, and RVLS strongly correlated with CMR values, suggesting that 3D-STE is a feasible and accurate alternative for RV function quantification [33]. However, 3D-STE has several limitations, including variability in vendor algorithms that require individual validation, reliance on optimal image quality for accurate tissue tracking, and lower frame rates, which can reduce accuracy. Additionally, there are wide limits of agreement with CMR, showing difficulties in aligning regional and slice-specific measurements between the two modalities. These limitations highlight the need for further validation before implementation in clinical practice [93].

### 15.2. Cardiac Magnetic Resonance

CMR can provide a comprehensive RV assessment via chamber quantification and strain analysis [147]. CMR-derived RV strain analysis is an emerging quantitative technique providing insights into myocardial motion and deformation beyond traditional parameters [148]. It can be done by feature tracking (FT) using conventional cine imaging, but dedicated acquisition methods such as strain-encoded imaging (SENC) or displacement encoding with stimulated echoes (DENSE) are newer techniques developed specifically for deformation assessment [149]. CMR-feature tracking (CMR-FT) has demonstrated significant potential in diagnosing and monitoring various clinical conditions, including arrhythmogenic right ventricular cardiomyopathy (ARVC) and ischemic mitral regurgitation (iMR). RV strain parameters can predict major adverse cardiac events (MACEs) and provide prognostic value beyond ejection fraction (EF) [124,148,150]. Endorsed by major cardiovascular imaging societies, guidelines emphasize the utility of CMR-derived strain metrics in assessing myocardial motion and predicting outcomes and exercise tolerance in conditions such as congenital heart disease [151]. Although CMR and TTE assess RV strain using different techniques that are not directly comparable, a head-to-head comparison of RVLS via STE and strain derived by CMR-FT showed moderate inter-modality correlation and incremental value in RV strain assessment [152]. Yet, CMR-derived RV strain has several limitations. It has lower temporal resolution than echo-based techniques, which can decrease accuracy. Standardized protocols and optimized imaging parameters are essential for reliable CMR-FT analysis [148,149,153,154], which can be affected by technical factors such as the CMR field strength and image quality. Additionally, strain-encoded sequences require dedicated post-processing models. Large-scale studies are required to establish standardized protocols and confirm the clinical utility of CMR-derived RV strain in different clinical indications. Furthermore, CMR has more limited access than TTE; while strain by CMR may provide additional insights into subclinical RV abnormalities, TTE remains the first line in RV assessment.

### 15.3. Artificial Intelligence (AI)

Current studies on the application of Artificial Intelligence (AI) in assessing right ventricular (RV) strain have shown promising results in improving the accuracy and efficiency of RV function evaluation. Liu et al. investigated the use of AI to evaluate RV function via echocardiographic parameters, focusing on region-specific strain using a vendor-neutral platform. The results demonstrated strong correlations between RV strain values and conventional echocardiographic parameters, such as FAC and TAPSE. The findings suggest that AI-based strain analysis can objectively assess RV function across different platforms, providing a reliable and time-efficient alternative to traditional methods [155].

Pense et al. showed that 3D Auto RV can simultaneously capture multiple 2DE parameters, including FAC, TAPSE, RVGLS, and RVLS. Although the differences in RVLS, TAPSE, and FAC obtained via this automated 3DE method were statistically significant compared with the conventional 2DE technique in 203 subjects (122 healthy and 81 cardiac patients), the differences were minimal in real terms [156]. Zhu et al. demonstrated that AI-based 3DE can provide rapid and accurate quantification of RV volumes and function, including strain measurements, which are crucial for identifying RV dysfunction. This study found significant correlations between AI-based 3DE and CMR-derived parameters, although AI-based 3DE slightly underestimated these parameters [157].

Additionally, Shad et al. demonstrated the application of a video AI system trained on preoperative echocardiography to predict postoperative RV failure. The system utilized the full spatiotemporal density of echocardiographic data, achieving an area under the curve (AUC) of 0.729, significantly outperforming human experts in independent evaluations [158]. These promising results highlight the potential of AI to predict RV dysfunction based on strain data from various imaging modalities, enhancing clinical decision making and improving patient outcomes [158].

## 16. Conclusions

RV strain imaging has emerged as a promising diagnostic modality in cardiovascular medicine, as it can provide enhanced sensitivity for detecting subtle myocardial dysfunction compared to conventional RV functional indices. Echocardiography, particularly 2D-STE, has been established as the preferred method for clinical assessment of RVLS. Recent advances and the standardization of its methodology have improved inter-vendor and inter-technique reliability. RVLS provides incremental diagnostic and prognostic value in various cardiac conditions affecting the RV. In particular, it may be useful for long-term follow-ups of chronic conditions such as pulmonary hypertension, pulmonary embolus, heart failure, and in cardio-oncology. However, accurate interpretation requires an understanding of RV mechanics, different cardiac pathologies, and the effects of technical factors such as tracking algorithms and imaging modalities. Three-dimensional imaging and AI advances have significant potential for expanding the clinical utility and precision of RV strain imaging. Further research is essential to determine its clinical applications and definitive role in the comprehensive evaluation and management of various cardiovascular diseases.

## Figures and Tables

**Table 1:** Summary of reference ranges for RVLS derived from RV-focused view using 2D-STE.

Study	Study Type	Sample Size	Ultrasound Device (s)	Strain Analysis Software	RVLS%		RVGLS%	
					Average	LLN	Average	LLN
Fine 2013 [34]	Prospective Cohort	186	Philips iE33, GE Vivid 7, Siemens Sequoia C512	Syngo VVI	−21.7	−13.3	−13.3	−14.0
Chia 2014 [35]	Prospective Cohort	142	GE Vivid 7	EchoPac	−27.3	−20.7	−22.4	−17.6
Morris 2016 [30]	Prospective Cohort	238	GE Vivid 7	EchoPac	−28.5	−18.9	−24.5	−16.9
Muraru 2016 [25]	Prospective Cohort	250	GE Vivid E9	EchoPac	−30.5	−22.7	−25.8	−19.8
McGhie 2017 [36]	Prospective Cohort	147	Philips iE33 or EPIQ7	TomTec	−25.4	−15.4	-	-
Park 2018 [37]	Prospective Cohort	493	GE	EchoPac	−26.4	−18.0	−21.5	−15.1
Addetia 2021 [31]	Retrospective Cohort	1913	Philips, Siemens, GE	TomTec	−28.3	−20.0	−25.4	−18.2
Wang 2021 [32]	Meta-analysis	3673	-	-	−26.9	−18.0	−23.4	−16.4
Espersen 2024 [38]	Prospective Cohort	2951	GE Vivid 9	EchoPac	−26.7	-	-	-

RVLS: right ventricular longitudinal strain; RVGLS: right ventricular global longitudinal strain; LLN: lower limit of normal.
